# Quality of health care in Germany: results of a pilot study to assess health system performance

**DOI:** 10.1093/eurpub/ckac131.025

**Published:** 2022-10-25

**Authors:** P Hengel, K Achstetter, M Blümel, V Schwarzbach, R Busse

**Affiliations:** Department of Health Care Management, Technische Universität Berlin, Berlin, Germany

## Abstract

**Background:**

Health System Performance Assessment (HSPA) is used as a tool to monitor and evaluate the performance of health systems and to inform evidence-based policymaking. For the first time, a systematic HSPA was piloted for Germany. The conceptual framework includes different dimensions, e.g., access, population health, efficiency, and quality of care. In the following, Germany’s performance is analysed in terms of quality of care.

**Methods:**

Indicators to assess the dimension of quality of care were selected based on a systematic search of established instruments in national and international HSPA initiatives. Other criteria for the inclusion of indicators were data availability and international comparability. The indicators were evaluated in terms of their time trend (2000-2020) and in international comparison (e.g., Austria, Denmark, France).

**Results:**

Overall, 17 indicators were selected to assess quality of care, of which two could not be analysed due to missing data. Indicators include, e.g., emergency readmissions after hospital stays, patient-reported medical errors, coercive measures in psychiatric wards, and in-hospital mortality. Trend analyses were possible for 14 indicators and most of them showed positive developments. In country comparisons, which were feasible for seven indicators, Germany mostly ranked below average. In-hospital mortality for acute myocardial infarction, e.g., was 8% in 2019 in Germany (other countries: 4%-7%) and has been stable since 2014. For stroke, Germany performs better and ranks three of five (6%; range: 5%-9%) in 2019.

**Conclusions:**

Measuring quality of care for a systematic and comparative German HSPA was proven to be feasible. However, some indicators could not be mapped so far due to lack of data. The results give insights into quality measurements across different sectors and can support evidence-based policymaking.

**Key messages:**

• In the first health system performance assessment (HSPA) for Germany, quality of care was evaluated over time (2000-2020) and compared to eight European countries using 17 indicators.

• Measurement of quality was feasible, but data availability should be strengthened in the future as country comparisons were possible for only half the indicators and two could not be analysed at all.

